# Effect of Different Initial Fermentation pH on Exopolysaccharides Produced by *Pseudoalteromonas agarivorans* Hao 2018 and Identification of Key Genes Involved in Exopolysaccharide Synthesis via Transcriptome Analysis

**DOI:** 10.3390/md20020089

**Published:** 2022-01-20

**Authors:** Yuhao Ju, Kai Shan, Wenlin Liu, Chenxiang Xi, Yiling Zhang, Wei Wang, Chunlei Wang, Ruiwen Cao, Wenxing Zhu, Haiyong Wang, Yanqiu Zhao, Lujiang Hao

**Affiliations:** 1 School of Bioengineering, Qilu University of Technology (Shandong Academy of Sciences), Jinan 250353, China; 10431201111@stu.qlu.edu.cn (Y.J.); kai_shan@163.com (K.S.); 1043118323@stu.qlu.edu.cn (W.L.); 1043119587@stu.qlu.edu.cn (C.X.); 10431200980@stu.qlu.edu.cn (Y.Z.); 10431211258@stu.qlu.edu.cn (W.W.); chunlei_wang79@163.com (C.W.); rwcao@qlu.edu.cn (R.C.); wenxingzhu@qlu.edu.cn (W.Z.); wangchemical@126.com (H.W.); 10431211120@stu.qlu.edu.cn (Y.Z.); 2 State Key Laboratory of Biobased Material and Green Papermaking, Qilu University of Technology (Shandong Academy of Sciences), Jinan 250353, China

**Keywords:** marine bacteria, structural analysis, transcriptome sequence, KEGG analysis, GO analysis

## Abstract

Exopolysaccharides (EPSs) are carbohydrate polymers produced and secreted by microorganisms. In a changing marine environment, EPS secretion can reduce damage from external environmental disturbances to microorganisms. Meanwhile, EPSs have promising application prospects in the fields of food, cosmetics, and pharmaceuticals. Changes in external environmental pH have been shown to affect the synthesis of EPSs in microorganisms. In this study, we analyzed the effects of different initial fermentation pHs on the production, monosaccharide composition, and antioxidant activity of the EPSs of *Pseudoalteromonas agarivorans* Hao 2018. In addition, the transcriptome sequence of *P. agarivorans* Hao 2018 under different initial fermentation pH levels was determined. GO and KEGG analyses showed that the differentially expressed genes were concentrated in the two-component regulatory system and bacterial chemotaxis pathways. We further identified the expression of key genes involved in EPS synthesis during pH changes. In particular, the expression of genes encoding the glucose/galactose MFS transporter, phosphomannomutase, and mannose-1-phosphate guanylyltransferase was upregulated when the environmental pH increased, thus promoting EPS synthesis. This study not only contributes to elucidating the environmental adaptation mechanisms of *P. agarivorans*, but also provides important theoretical guidance for the directed development of new products using biologically active polysaccharides.

## 1. Introduction

Exopolysaccharide (EPS) secretion is essential for marine microbes. EPSs play an important role in promoting the formation of biological aggregates [1,2,3], enhancing the adhesion of microorganisms [4,5,6,7], promoting the formation of biofilms [8,9], absorbing surrounding nutrients [10], providing protective barriers [11,12], and maintaining the stability of ecosystems [13,14]. In addition, EPSs from marine bacteria have marked anti-tumor, antiviral, and immunomodulatory functions [15]. Previous studies have shown that the exopolysaccharide produced by *P. agarivorans* Hao 2018 has good antioxidant activity, hygroscopicity, and moisture retention, and also has good absorption of Cu^2+^ and Pb^2+^ [16,17].

The pH of the fermentation medium is one of the most critical environmental parameters for bacterial exopolysaccharide biosynthesis. However, the effects of pH on the biosynthesis of exopolysaccharides and cell growth are also different due to the types of microorganisms, operating conditions, and medium composition [18,19,20,21]. Studies have shown that changes in pH will affect the molecular weight and production of bacterial exopolysaccharides. Shu and Lung found that when the initial pH of flask cultures was adjusted from 3.0 to 6.0, the distribution of the molecular weight of exopolysaccharide shifted from higher molecular weights toward lower molecular weights and number-average molecular weight decreased monotonically from 7.98 × 10^5^ to 2.18 × 10^5^ [22]. Fang et al. reduced the initial pH of *Ganoderma lucidum* fermentation from 6.5 to 3.5, and found that the yield of EPSs increased [23]. In addition, using RNA-Seq technology, Hu et al. found that *Escherichia coli* 0157:H7 synthesized more antioxidant enzymes to repair the damage caused by acid and to enhance its resistance to acid [24].

In this study, the EPS-producing marine bacterium *P. agarivorans* was derived from the microbial membrane on the surface of abalone seedlings. Based on the study by Hao et al., the strain *P. agarivorans* Hao 2018 was cultured by changing the initial pH of the fermentation, and then EPSs were obtained by extraction, isolation, and purification to study the effect of environmental disturbances on EPS [17,25]. The monosaccharide composition of EPS was analyzed via high-performance liquid chromatography (HPLC) and Fourier transform infrared (FT-IR) spectroscopy. In addition, the scavenging ability of EPSs against hydroxyl radicals, DPPH radicals, and ABTS radicals was used as an index to evaluate their antioxidant activity. Finally, transcriptome sequencing of *P. agarivorans* Hao 2018 after fermentation at different initial pH levels was performed, and differential gene expression was studied to identify important functional genes and EPS synthesis genes, as well as to analyze the molecular regulatory mechanisms of EPS synthesis and the regulatory mechanisms of *P. agarivorans* Hao 2018 in response to environmental pH perturbation.

## 2. Results

### 2.1. Analysis of the Monosaccharide Composition of EPSs via HPLC

**Figure 1:**
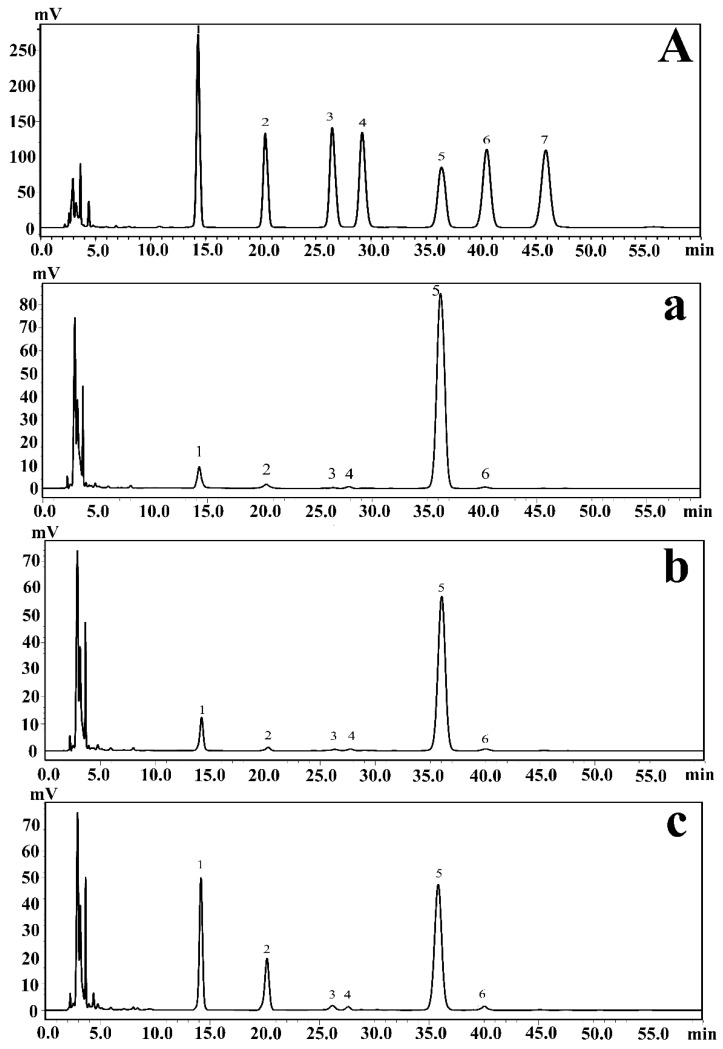
(**A**) HPLC chromatograms of seven PMP-labeled standard monosaccharides. (**a**–**c**) HPLC chromatograms of PMP-labeled monosaccharides released from exopolysaccharides under different pH conditions; (**a**–**c**) represent the results at pH 7, pH 8, and pH 9, respectively. Peaks: 1. Mannose; 2. Rhamnose; 3. Glucuronic acid; 4. Galacturonic acid; 5. Glucose; 6. Galactose; 7. Arabinose.

Monosaccharide composition analysis could help determine the types and amounts of multiple monosaccharides in carbohydrates in EPSs. Furthermore, this information plays a crucial role in the analysis of the regulation of EPSs produced by *P. agarivorans* Hao 2018. The EPSs produced under different initial fermentation pH values were hydrolyzed by sulfuric acid and then derivatized with PMP (1-phenyl-3-methyl-5-pyrazolinone). Using HPLC analysis, the monosaccharide composition of the EPSs produced by *P. agarivorans* Hao 2018 was identified as mannose, rhamnose, glucuronic acid, galacturonic acid, glucose, and galactose. The main components of the EPSs were glucose, mannose, and rhamnose. As the initial fermentation pH increased from 7 to 9, the proportion of mannose also increased from 6.66% to 25.72%, whereas the proportion of glucose decreased from 90.28% to 54.99% (Figure 1).

### 2.2. Analysis of the EPSs via FT-IR

**Figure 2:**
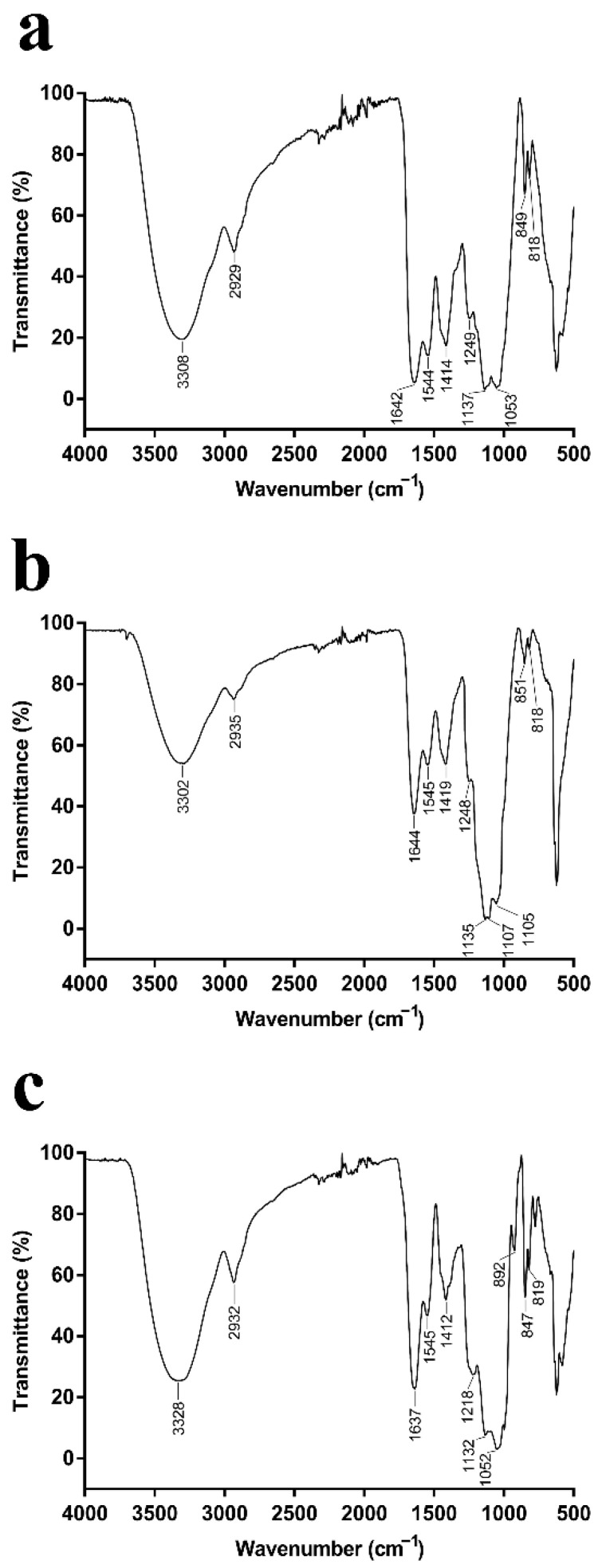
(**a**–**c**) FT-IR spectra of the purified EPSs from *P. agarivorans* Hao 2018 at pH 7, pH 8, and pH 9, respectively. All the FT-IR analysis of EPS fractions were performed in the region 400–5000cm^−1^.

When IR radiation interacts with molecules or compounds, their chemical bonds or functional groups vibrate at different frequencies, thus allowing the identification of the chemical bonds or functional groups in the molecule by the location of the peaks [26]. The EPSs produced by *P. agarivorans* Hao 2018 at different initial fermentation pHs exhibited features that are characteristic of polysaccharides, including hydroxyl group bands, alkyl group bands, and carboxyl group bands (Figure 2) [27,28,29,30]. The strong and broad absorption peaks at 3308 cm^−1^, 3302 cm^−1^, and 3328 cm^−1^ were characteristic of O-H groups. The peaks at 2929 cm^−1^, 2935 cm^−1^, and 2932 cm^−1^ indicate the presence of weak C-H bond stretching vibrations, and the peaks at 1642 cm^−1^, 1644 cm^−1^, and 1637 cm^−1^ are the C=O stretching vibrations of the -CHO group. The peaks within the range of 1000–1200 cm^−1^ suggested the presence of C-O-C and C-O-H bonds, indicating the presence of pyranose rings [31]. Moreover, the α and β configurations can be clearly distinguished by the anomeric region-vibrational bands in the 950 to 750 cm^−1^ region, where 847–851 cm^−1^ (Figure 2a–c) corresponds to the α configuration, while the β configuration lies around 892 cm^−1^ (Figure 2c) [32,33].

### 2.3. Analysis of the Antioxidant Activity of EPSs

**Figure 3:**
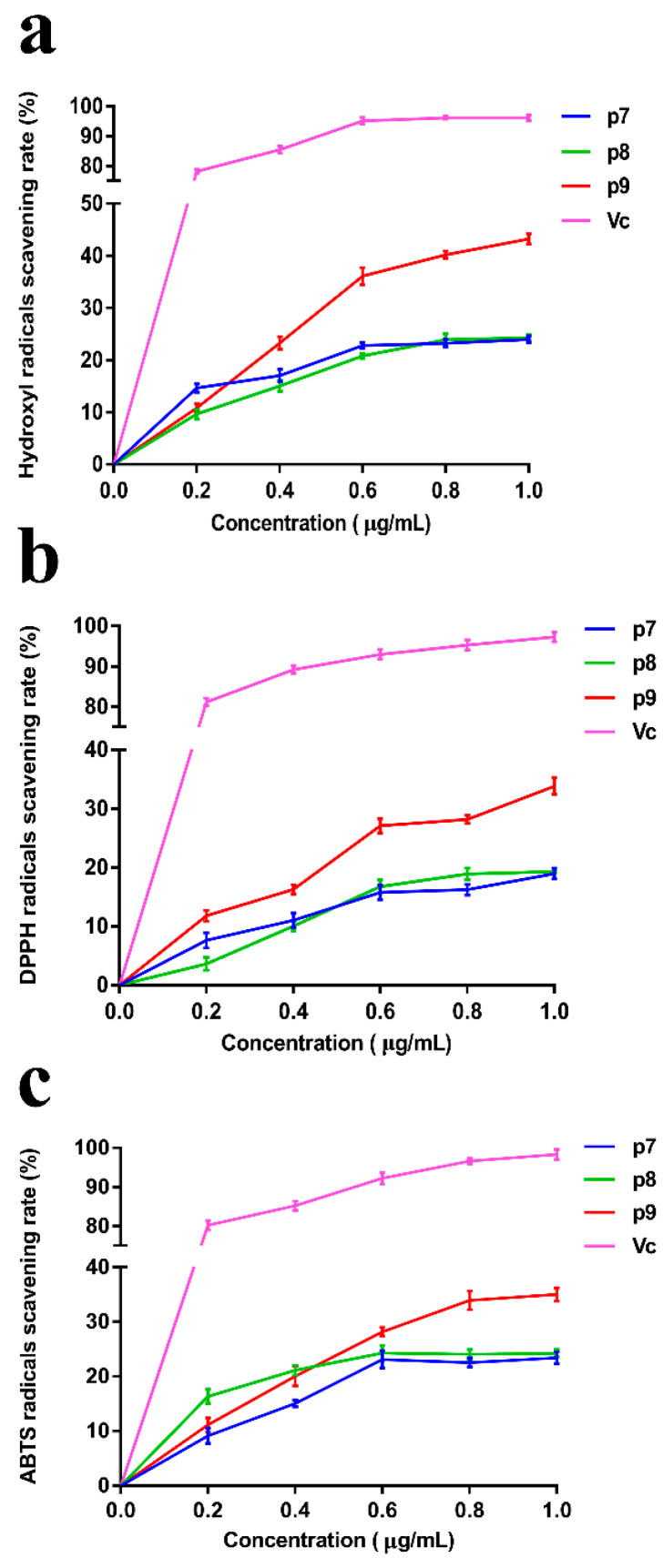
The scavenging effect of EPSs on (**a**) Hydroxyl radicals, (**b**) DPPH radicals, and (**c**) ABTS radicals. Vitamin C (Vc) was used as a control.

In recent years, with the increasing knowledge and research on free radicals, the exploration of efficient and non-toxic free radical scavengers has become a popular topic in biochemistry and medicine. Meanwhile, the development of natural active substances from the sea has made great progress, and a large number of studies have shown that marine polysaccharides have good antioxidant effects. In this study, the scavenging ability of *P. agarivorans* Hao 2018 EPSs against hydroxyl radicals, DPPH radicals, and ABTS radicals under different fermentation initial pH conditions was evaluated to compare its antioxidant activity (Figure 3). It can be seen that although the scavenging rate of EPS on the three free radicals is lower than that of Vitamin C (Vc), it has a certain scavenging effect. The antioxidant activity of EPSs under different pH conditions gradually increased with increasing concentrations. The highest scavenging rates of hydroxyl, DPPH, and ABTS radicals were determined at the initial fermentation pH of 9 when EPSs were at the highest concentration (1 μg/mL). At an EPS concentration of 1 mg/L, the extracellular polysaccharides produced under the condition of an initial fermentation pH of 9 have the most significant scavenging effect on hydroxyl free radicals, and the scavenging rate was 43.24%.

### 2.4. Sequencing of the Pseudoalteromonas agarivorans Hao 2018 Transcriptome

**Figure 4:**
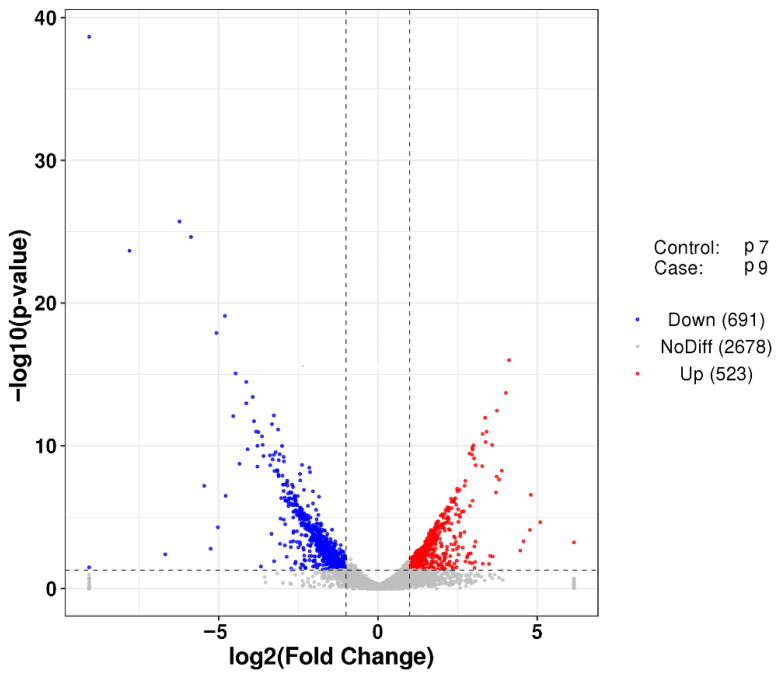
Volcano plot of differentially expressed genes between group P7 and group P9. The vertical line is twice of the expression difference threshold, and the horizontal line represents the *p*-value = 0.05. Red dots represent significantly upregulated genes and blue dots represent significantly downregulated genes (*p*-value < 0.05). Gray dots indicate non-significant differentially expressed genes.

After transcriptome sequencing, the image files were transformed using the Illumina MiSeq system. Three biological replicates used in each group (P7 and P9) produced more than 2.02 × 10^8^ raw reads, and the raw reads were filtered to remove some spliced, low-quality sequences, obtaining approximately 1.89 × 10^8^ clean reads. The proportion of high-quality sequences and bases of the two groups of samples was above 93.22%. The quality of sequencing data was assessed using FastQC software, and the criteria included base quality distribution, base content distribution, and average sequence quality distribution, and the evaluation results, all of which showed that the data were accurate and valid (Figure S1). Samples P7 and P9 had mean sequence quality over 25, indicating that the mean quality and mean quality distribution of both sequencing datasets were better (Figure S2). FPKM (fragments per kilobase million) density distribution provides an overall look at the gene expression patterns of the samples, with moderately expressed genes accounting for the majority of the samples and a small percentage of low- and high expression genes (Figure S3). Finally, the differences in gene expression of *P. agarivorans* Hao 2018 in the two groups (P7, P9) were analyzed using the DESeq software package in the R programming language. The results showed that 523 genes were upregulated and 691 genes were downregulated in the P9 group compared to the P7 group (Figure 4).

### 2.5. GO and KEGG Analysis to Identify the Metabolic Processes Involving Genes with Significant Differences

**Figure 5:**
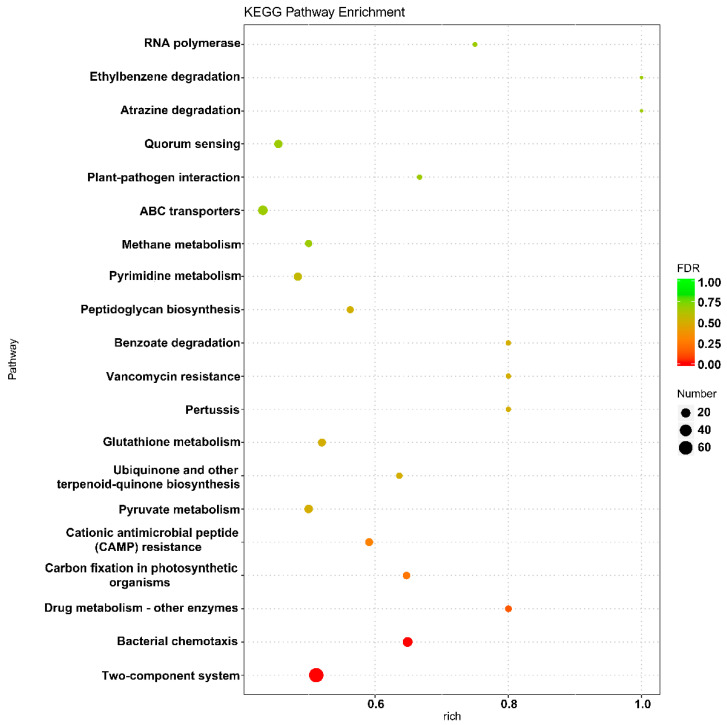
Scatterplot of enriched KEGG pathways for differentially expressed genes in group P7 and group P9. The X-axis represents the percentages of differentially expressed genes belonging to the corresponding pathway, and the Y-axis represents the top twenty pathways. The size of a bubble represents the number of differentially expressed genes in the corresponding pathway, and the colors of the bubble represent the enrichment *p*-value of the corresponding pathway.

The results of GO enrichment analysis showed that the differentially expressed genes in the P7 and P9 groups were primarily involved in molecular biofunctional classifications, such as molecular transducer activity, receptor activity, and biological process classifications, such as signal transduction, cell communication, cell cycle, and nucleoside phosphate biosynthetic processes. The enrichment of genes for nucleobase-containing compound biosynthetic processes, heterocycle biosynthetic processes, and aromatic compound biosynthetic processes were also significant (Table S2). In addition, KEGG enrichment analysis showed that differentially expressed genes were primarily enriched in the two-component regulatory system and bacterial chemotaxis pathways (Figure 5).

### 2.6. Analysis of the Expression of Genes Related to the Two-Component Regulatory System

**Figure 6:**
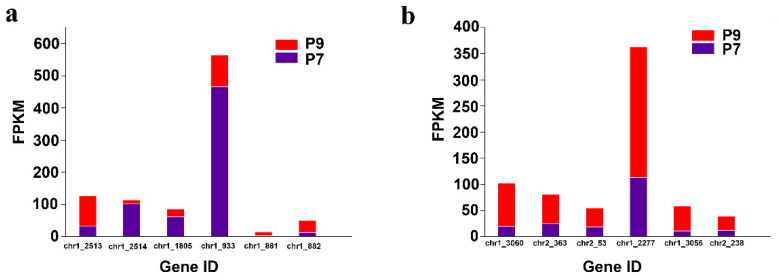
Differential expression of genes related to the two-component regulatory system and bacterial chemotaxis. (**a**) Two-component regulatory system related gene expression. (**b**) Bacterial chemotaxis related gene expression.

The two-component regulatory system is a signal transduction mechanism commonly found in various prokaryotes and a few eukaryotes, which indirectly affects EPS synthesis to help microorganisms survive better in different environments by sensing changes in external signals and stimulating cells to respond adaptively to external environmental perturbations. The two-component regulatory system generally consists of two basic components: histidine kinase and response-regulated protein (Figure S4 [34]). Bacteria generally possess multiple two-component regulatory systems that regulate perturbations in the external environment. In addition, bacterial chemotaxis can be regulated by a two-component regulatory system. Examples of common two-component regulatory systems are *PhoR/PhoB, PhoQ/PhoP, RstB/RstA, BaeS/BaeR, EnvZ/OmpR, QseC/QseB, CreC/CreB, NtrB/NtrC,* and *CheA/CheY* [35,36,37,38,39,40,41]. In this study, compared with the P7 group, the genes encoding histidine kinases *PhoR* (chr1_2513), *BaeS* (chr1_881), and *CheA* (chr1_3060), the gene encoding the response regulator protein *BaeR* (chr1_882), *CheY* (chr2_363, chr2_53), *CheB* (chr1_2277, chr1_3055), and *WspR* (chr2_238) were upregulated, and the genes encoding histidine kinase *PhoQ* (chr1_1805), the gene encoding the response regulator protein *PhoB* (chr1_2514), and *RstA* (chr1_933) were downregulated in the P9 group (Figure 6a,b).

### 2.7. Analysis of the Expression of Genes Directly Related to EPS Synthesis

**Figure 7:**
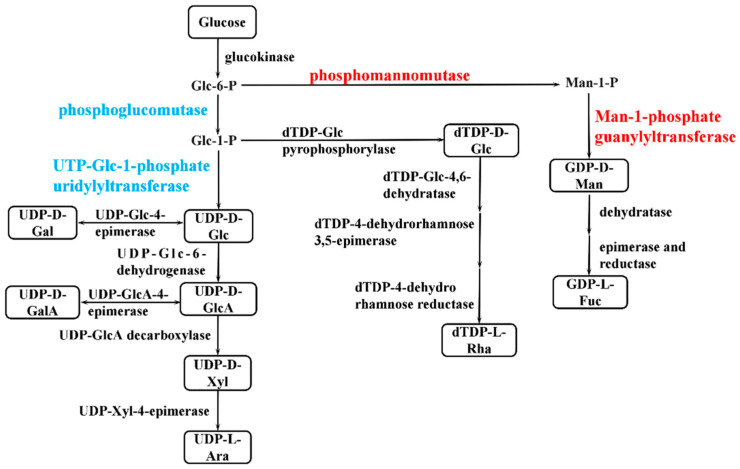
Simplified pathway of sugar nucleotides synthesis in *P. agarivorans* Hao 2018. Sugar nucleotides are shown in boxes and key enzymes of each pathway are indicated in red (upregulated), blue (downregulated) and black (no significant differences).

**Figure 8:**
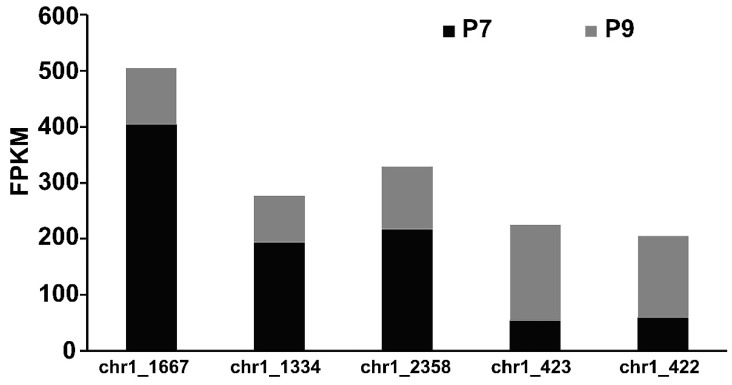
Expression of key genes related to nucleotide sugar synthesis.

The synthesis of bacterial EPSs requires the involvement of multiple classes of genes, including transport-related genes, nucleotide sugar synthesis-related genes, and genes encoding glycosyltransferase. The expression of genes related to glucose transporter proteins (chr1_1825, chr2_538, chr1_1082) was higher in the P9 group than in the P7 group (Table S3). When *P. agarivorans* Hao 2018 used glucose as a carbon source for EPS synthesis, Chr1_1825, chr2_538, and chr1_1082, which can regulate the transport of glucose by monosaccharide transport proteins into the cell for cellular use. After glucose enters the cell, it is first transformed by glucokinase to produce glucose-6-phosphate and then converted into the corresponding nucleotide sugars through the monosaccharide phosphorylation reaction, as shown in Figure 7. In the P9 group, the expression of genes encoding UTP-glucose-1-phosphate uridylyltransferase (chr1_1334, chr1_2358, and chr1_459) was lower than that in the P7 group, and the expression of genes encoding phosphomannomutase (chr1_423) and mannose-1-phosphate guanylyltransferase (chr1_422) was higher than that in the P7 group (Figure 8 and Table S4), which was consistent with the results of the monosaccharide ratio determined by monosaccharide composition. After the synthesis of nucleotide sugars, the main and side chains of the repeating units are formed under the action of glycosyltransferases. A total of 17 genes related to glycosyltransferase synthesis were identified in *P. agarivorans* Hao 2018. Compared with the P7 group, seven genes were differentially expressed in the P9 group, four genes were upregulated, and three genes were downregulated (Table S5).

## 3. Discussion

More and more biological activities of exopolysaccharides have been demonstrated, and these activities are related to the chemical composition, configuration, and physical properties of the polysaccharides. Although the structure-activity relationships of complex exopolysaccharides are difficult to elucidate, some possible relationships can be inferred. Many polysaccharides and their derivatives scavenge free radicals effectively and can be used as antioxidants [42]. Lo et al. found that the antioxidant activity of *Lentinula edodes* polysaccharides increased with an increase in the ratio of mannose and rhamnose and decreased with an increasing ratio of arabinose and glucose [43]. The high percentage of mannose in the polysaccharides produced by *Hirsutella* sp. had a positive effect on their biological activity [44]. Our results are in good agreement with the above report. The antioxidant activity of EPSs increased with an increase in the initial pH of fermentation, which might be related to the proportion of mannose in the monosaccharide composition of EPSs produced when the initial fermentation pH was 9. Moreover, it can be seen from the growth curve (Figure S5) that the strain *P.* *agarivorans* Hao 2018 has a better growth trend when the initial fermentation pH was 8, which is consistent with the results of the previous study. Interestingly, the growth trend of strain *P.* *agarivorans* Hao 2018 was better at an initial fermentation pH of 9 than at pH 7. Combined with the crude yield of exopolysaccharides (Table S6) of *P.* *agarivorans* Hao 2018 at different initial fermentation pH values, we hypothesized that strain *P.* *agarivorans* Hao 2018 would resist the high pH perturbation by secreting exopolysaccharides. In the present study, we also analyzed the effect of EPSs produced by *P.* *agarivorans* Hao 2018 under different initial fermentation pH conditions at the molecular level. Our results indicate that key genes (Chr1_1825, Chr2_538, Chr_423, Chr_422, chr2_238, etc.) associated with *P. agarivorans* Hao 2018 EPS synthesis showed upregulated expression when the environmental pH was elevated. The results of HPLC analysis of EPS monosaccharide composition change coincided with the expression of genes related to nucleotide sugar synthesis. To date, many marine bacteria that can secrete EPSs have been isolated, including members of *Acinetobacter*, *Arthrobacter*, *Pseudomonas*, *Halomonas*, *Myroides*, *Corynebacteria*, and *Bacillus* [45]. However, scientists have mostly focused on the structural elucidation and fermentation process control of highly biologically active marine microbial polysaccharides, but the molecular regulatory mechanisms of EPS synthesis by environmental perturbations still need to be explored, especially for the exploration of key genes in the EPS synthesis pathway. Marzan et al. found that *E. coli phoB* and *phoR* gene knockout mutant strains increased the rate of glucose and gluconate uptake compared to wild strains [46], and Sultan et al. also found that *ΔphoB* El Tor *Vibrio cholerae* mutant strains had significantly higher expression levels of EPS synthesis genes *vpsA* and *vpsL*, which promote biofilm formation [47]. In this study, the gene encoding the response-regulated protein *phoB* (chr1_2514) was downregulated with an increase in the initial fermentation pH, thereby negatively regulating biofilm formation. In addition, the synthesis of most bacterial EPSs is regulated by cyclic diguanylate (c-di-GMP), a second messenger involved in the regulation of various physiological functions of bacteria. Hickman et al. [48]. found that in *Pseudomonas aeruginosa, WspF* is a homologous gene of the chemotactic gene *CheB*. The deletion of *WspF* leads to an increase in the level of intracellular c-di-GMP, which promotes biofilm formation and EPS synthesis. These phenotypes depend on the existence of the response regulator of the two-component regulatory system, *WspR*, which contains the GGDEF structural domain that is phosphorylated to enhance the catalytic activity of diguanylate cyclase (DGCs), and *WspR* is phosphorylated to enhance the synthesis of c-di-GMP. Our results show that the gene expression corresponding to *WspR* (chr2_238) was upregulated when the environmental pH was increased, thus promoting EPS synthesis. The increase in environmental pH upregulates the expression of genes encoding phosphomannomutase (chr1_423) and mannose-1-phosphate guanylyltransferase (chr1_422), thus increasing the synthesis of mannose, which makes it possible to develop new products of polysaccharides with biological activity. Although the EPS synthesis pathway of *P. agarivorans* Hao 2018 has not been clarified, the gene mining of key enzymes in the nucleotide sugar synthesis pathway in this study provides the basis for subsequent studies.

## 4. Materials and Methods

### 4.1. Extraction and Purification of EPSs under Different Fermentation Conditions

Marine bacterial cells of *P. agarivorans* Hao 2018 were transferred to Zobell 2216E liquid medium (peptone 5 g/L, yeast extract 1 g/L, sea salt 35 g/L, pH 8) and cultured for 8 h at 25 °C, 180 rpm in a shaker; 8% of the seed volume was transferred to the fermentation medium (glucose 30 g/L; yeast extract 4.5 g/L, sea salt 35 g/L) under different culture conditions (Table S1). The fermentation broth was shaken for 36 h at 15 °C and then centrifuged at 4 °C and 4000 rpm for 5 min to remove the bacteria; the supernatant was poured into a rotary evaporator, concentrated to one-third of the original volume, and three times the volume of 95% ethanol was slowly added to it while stirring with a magnetic stirrer, after which it was placed in a refrigerator at 4 °C overnight for complete precipitation. The mixture was centrifuged at 4 °C and 8000 rpm for 10 min. The organic solvent and denatured proteins in the precipitates were removed using Savage reagent (chloroform: *n*-butanol = 5:1) [49]. The total sugar content of EPS was determined by the phenol-sulfuric acid method. The polysaccharides were formed into sugar-aldehyde derivatives under the function of sulfuric acid, which in turn formed an orange-yellow compound with phenol, and then determined using a colorimetric method.

The EPS in deionized water was further purified by using DEAE-52 anion-exchange chromatography and gel filtration chromatography [50]. Then, the purified EPS was dialyzed with deionized water and selective semipermeable membranes (8000~14,400 da) [17]. The polysaccharides were freeze-dried and stored at 4°C, and labeled as P7, P8, and P9, respectively.

### 4.2. Determination of EPSs via HPLC and FT-IR

The 1-phenyl-3-methyl-5-pyrazolinone (PMP) pre-column derivatization method is a widely used and convenient method for the determination of exopolysaccharides. Pure EPS (25 mg) was weighed under different fermentation conditions, poured into 10 mL of 1 mol/L H_2_SO_4_, and hydrolyzed in a water bath at 100 °C for 8 h. After centrifugation at 8000 rpm for 5 min, 2 mol/L of NaOH was added to the supernatant to obtain a final pH of 7. Thereafter, 5 mL of hydrolysis solution was mixed with 5 mL of deionized water to obtain a solution for EPS hydrolysis. 50 μL of EPS hydrolysis solution was mixed with 50 μL of PMP methanol solution (0.5 mol/L) and 50 μL of NaOH (0.3 mol/L) solution and reacted in a constant temperature water bath at 70 °C for 30 min. After cooling to 25 °C, 50 μL of HCl (0.3 mol/L) was added to neutralize the mixture, and then 100 μL of deionized water was added to dilute it. Next, 900 μL of trichloromethane was added and the mixture was extracted three times, centrifuged at 4000 rpm for 5 min, and then the lower turbid layer was discarded. The supernatant was filtered through a 0.22 μm filter membrane, and the aqueous phase was prepared for use. All tests were conducted independently three times. For HPLC, the mobile phase consisted of ammonium acetate buffer solution (pH 5.5) and acetonitrile in a volume ratio of 80:20, with a flow rate of 1 mL/min, column temperature of 30 °C, and EPS detection at a wavelength of 245 nm. The FT-IR spectra of the samples were recorded using an FT-IR spectrometer (PerkinElmer, Norwalk, CT, USA) [51].

### 4.3. Determination of Antioxidant Activity of EPSs to Scavenge OH, DPPH, and ABTS Free Radicals

The Fenton reaction determines the scavenging rate of the sample to -OH by measuring the OD value of the product generated by the reaction between salicylic acid and -OH. After 0.1 mL FeSO_4_ solution (9 mmol/L), 0.1 mL salicylic acid-ethanol solution (9 mmol/L), and different concentrations of EPSs (0.2, 0.4, 0.6, 0.8, 1 mg/mL) were added to 96-well plates, and then 0.1 mL 1 mmol/L H_2_O_2_ was added to form the reaction system. The reaction mixture was incubated at 37 °C for 30 min. Finally, the absorbance was measured at 510 nm [52].

$$\mathrm{OH}\;\mathrm{free}\;\mathrm{radical}\;\mathrm{scavenging}\;\mathrm{rate}\;\left(\%\right)=\left(1-\frac{A_{X1}-A_{1}}{A_{0}}\right)\times 100\%$$

Fifty microliters of different concentrations of EPS samples (0.2, 0.4, 0.6, 0.8, and 1 mg/mL) were added to the 96-well plates, followed by 25 μL of DPPH-ethanol solution (0.4 mmol/L) and 100 μL of deionized water. The system was mixed and allowed to react at 30 °C for 30 min without light. Finally, the absorbance was measured at 517 nm [53].

$$\mathrm{DPPH}\;\mathrm{free}\;\mathrm{radical}\;\mathrm{scavenging}\;\mathrm{rate}\;\left(\%\right)=\left(1-\frac{A_{X2}-A_{2}}{A_{0}}\right)\times 100\%$$

The ABTS working solution [54] was diluted with deionized water to an absorbance value of 0.70 ± 0.02 at 734 nm. Twenty microliters of different concentrations of EPS solutions of different concentrations (0.2, 0.4, 0.6, 0.8, and 1 mg/mL) were added to the 96-well plates, followed by 200 μL of diluted ABTS solution, mixed well, and incubated for 6 min at 25 °C. Finally, the absorbance was measured at 734 nm.

$$\mathrm{ABTS}\;\mathrm{free}\;\mathrm{radical}\;\mathrm{scavenging}\;\mathrm{rate}\;\left(\%\right)=\left(1-\frac{A_{X3}-A_{3}}{A_{0}}\;\right)\times 100\%$$

A_Xn_ indicates the absorbance values of different concentrations of EPS reaction solution, A_0_ represents the absorbance of blank solution, and A_n_ symbolizes the absorption of the EPS solution background. The tests were repeated three times and averaged.

### 4.4. Transcriptome Sequencing

The strain *P. agarivorans* Hao 2018 was inoculated into Zobell 2216E liquid medium and cultured at 25 °C and 180 rpm for 8 h, then transferred to a fermentation medium at an initial pH of 7 or an initial pH of 9 at 8% inoculum and cultured for 36 h at 15 °C and 180 rpm with constant temperature shaking. Bacteria were collected by centrifugation at 4 °C and 8000 rpm for 10 min after the culture, and labeled as P7 and P9, respectively. Three biological replicates were used for each group. The wholeRNA of the strain *P. agarivorans* Hao 2018 was extracted using the TRIzol kit, and the quality was checked using a NanoDrop spectrophotometer and Agilent 2100 Bioanalyzer. The rRNA was removed using the Ribo-Zero rRNA Magnetic Kit, and the mRNA was pre-treated using a Truseq TM RNA sample prep kit. The first strand of cDNA was synthesized using random primers and SuperScript III reverse transcriptase, and the second strand was synthesized by replacing dTTP with dUTP, after which the adaptor junction was attached, and the second strand of cDNA was digested by adding uracil-N-glycosylase. The sequencing process and preliminary analysis were performed by Shanghai Paisano Biotechnology Co.

### 4.5. Transcriptome Data Analysis

The Cutadapt software (Version 2.4) [55] was used to remove raw data with at least 10 bp overlap (AGATCGGAAG) at the 3′ end with a known junction, allowing 20% base mismatches, and also to remove reads with average quality scores below Q20. The quality of sequencing data was evaluated using FastQC software, and the evaluation criteria included base quality distribution, base content distribution, and average sequence quality distribution. The high-quality reads obtained after filtering and quality control were compared with the reference genome of *P. agarivorans* Hao 2018 using Bowtie2 [56] and Tophat2 [57] sequence comparison software. Raw gene expression was obtained by HTSeq 0.6.1p2 [58] compared to the number of reads for each gene, and FPKM values were used to normalize the expression of genes in the samples, thus making the gene expression levels comparable across genes or samples. Gene expression differences were analyzed using the R programming language DESeq software package [59] (version 1.18.0) with the screening conditions log2|fold change| > 1 and *p*-value < 0.05, and the screening results were visualized using the R programming language graphical visualization package ggplot2. The reference genome of *P. agarivorans* Hao 2018 was used as a background to analyze the biological functions of differentially expressed genes by calculating the *p*-value of GO terms that were significantly enriched (*p* < 0.05) for differential genes using a hypergeometric distribution method [60]. The number of differentially expressed genes at different levels of KEGG was counted using the analytical tool KOBAS to analyze the metabolic pathways in which the differentially expressed genes were primarily involved [61].

## 5. Conclusions

In this study, the monosaccharide composition and glycosidic bond configuration of the EPSs of *P. agarivorans* Hao 2018 were determined when produced under different pH conditions, and the effects of environmental perturbations on the structure of the EPSs were analyzed. HPLC results showed that the main components of EPSs were glucose, mannose, and rhamnose, and when the initial pH of fermentation increased, the proportion of mannose also increased, whereas the proportion of glucose decreased. FT-IR analysis showed that the EPSs produced during fermentation at an initial pH of 9 showed characteristic absorption peaks of β configurations. Meanwhile, EPSs produced at an initial fermentation pH of 9 showed the most significant scavenging of hydroxyl, DPPH, and ABTS radicals. Finally, we performed transcriptome sequencing of *P. agarivorans* Hao 2018 after fermentation at an initial pH of 7 and 9 and compared the differences in gene expression between the two groups, with 523 genes upregulated and 691 genes downregulated in the P9 group compared to the P7 group. The results of GO and KEGG enrichment analyses showed that the differential genes in the P7 and P9 groups were primarily enriched in the two-component regulatory system and bacterial chemotaxis pathway. We also determined the expression of key genes involved in EPS synthesis with pH change. Genes related to glucose transporter proteins, key enzymes in nucleotide sugar synthesis, and glycosyltransferases, which are directly related to EPS synthesis, showed upregulated expression at increased environmental pH levels. These interesting findings may provide useful insights into the molecular mechanism of extracellular polysaccharide synthesis in *P. agarivorans* Hao 2018. Furthermore, these findings provide information for the development of extracellular polysaccharide products with good biological activity.

## Data Availability

The data presented in this study are available on request from the corresponding author. The data are not publicly available due to these data also form part of an ongoing study.

## Supplementary Materials

The following are available online at https://www.mdpi.com/article/10.3390/md20020089/s1, Figure S1: Single base mass distribution chart; Figure S2: Reads average mass distribution (partial); Figure S3: FPKM density distribution and density distribution statistics: (a) FPKM density distribution, (b) FPKM density distribution statistical; Figure S4: Regulatory mechanism of the two-component system; Figure S5: The growth curves of *P. agarivorans* Hao 2018 under different initial fermentation pH conditions, Table S1: Culture conditions of *P. agarivorans* Hao 2018; Table S2: Results of GO enrichment analysis; Table S3: Expression of EPS transporter-related genes; Table S4: Expression of genes related to nucleotide sugar synthesis; Table S5: Expression of genes related to glycosyltransferase synthesis; Table S6: Effect of different initial fermentation pHs on the yield of crude EPS.

## References

[1] Harris R.H., Mitchell R. (1973). The role of polymers in microbial aggregation. Annu. Rev. Microbiol..

[2] Biddanda B. (1985). Microbial synthesis of macroparticulate matter. Mar. Ecol. Prog. Ser..

[3] Alldredge A.L., Silver M.W. (1988). Characteristics, dynamics and significance of marine snow. Prog. Oceanogr..

[4] Paerl H.W. (1975). Microbial attachment to particles in marine and freshwater ecosystems. Microb. Ecol..

[5] Marshall K.C., Savage D.C., Fletcher M. (1985). Mechanisms of bacterial adhesion at solid-water interfaces. Bacterial Adhesion.

[6] Vincent P., Pignet P., Talmont F., Bozzi L., Fournet B., Guezennec J., Jeanthon C., Prieur D. (1994). Production and characterization of an exopolysaccharide excreted by a deep-Sea hydrothermal vent bacterium isolated from the polychaete annelid *Alvinella pompejana*. Appl. Environ. Microbiol..

[7] Holmström C., Kjelleberg S. (1999). Marine *Pseudoalteromonas* species are associated with higher organisms and produce biologically active extracellular agents. FEMS Microbiol. Ecol..

[8] Sutherland I.W., Wingender J., Neu T.R., Flemming H.-C. (1999). Biofilm Exopolysaccharides. Microbial Extracellular Polymeric Substances.

[9] Sutherland I.W. (2001). Biofilm Exopolysaccharides: A strong and sticky framework. Microbiology.

[10] Decho A.W., Herndl G.J. (1995). Microbial activities and the transformation of organic matter within mucilaginous material. Sci. Total Environ..

[11] Bitton G., Freihofer V. (1977). Influence of extracellular polysaccharides on the toxicity of copper and cadmium Toward *Klebsiella aerogenes*. Microb. Ecol..

[12] Decho A.W., Lopez G.R. (1993). Exopolymer microenvironments of microbial flora: Multiple and interactive effects on trophic relationships. Limnol. Oceanogr..

[13] Dade W.B., Davis J.D., Nichols P.D., Nowell A.R.M., Thistle D., Trexler M.B., White D.C. (1990). Effects of bacterial exopolymer adhesion on the entrainment of sand. Geomicrobiol. J..

[14] Uhlinger D.J., White D.C. (1983). Relationship between physiological status and formation of extracellular polysaccha ride glycocalyx in *Pseudomonas atlantica*. Appl. Environ. Microbiol..

[15] Llamas I., Mata J.A., Tallon R., Bressollier P., Urdaci M.C., Quesada E., Béjar V. (2010). Characterization of the exopolysaccharide produced by *Salipiger mucosus* A3T, a Halophilic Species Belonging to the Alphaproteobacteria, Isolated on the Spanish Mediterranean Seaboard. Mar. Drugs.

[16] Wang C., Fan Q., Zhang X., Lu X., Xu Y., Zhu W., Zhang J., Hao W., Hao L. (2018). Isolation, characterization, and pharmaceutical applications of an exopolysaccharide from *Aerococcus uriaeequi*. Mar. Drugs.

[17] Hao L., Liu W., Liu K., Shan K., Wang C., Xi C., Liu J., Fan Q., Zhang X., Lu X. (2019). Isolation, optimization of fermentation conditions, and characterization of an exopolysaccharide from *Pseudoalteromonas agarivorans* Hao 2018. Mar. Drugs.

[18] Vinroot S., Torzilli A.P. (1988). Interactive effects of inoculum density, agitation, and Ph on dimorphism in a salt marsh isolate of *Aureobasidium pullulans*. Mycologia.

[19] Esgalhado M.E., Roseiro J.C., Collaço M.T.A. (1995). Interactive effects of pH and temperature on cell growth and polymer production by *Xanthomonas campestris*. Process Biochem..

[20] Yang F.-C., Liau C.-B. (1998). The influence of environmental conditions on polysaccharide formation by *Ganoderma lucidum* in submerged cultures. Process Biochem..

[21] Catley B.J. (1971). Role of pH and nitrogen limitation in the elaboration of the extracellular polysaccharide pullulan by *Pullularia pullulans*. Appl. Microbiol..

[22] Shu C.-H., Lung M.-Y. (2004). Effect of pH on the production and molecular weight distribution of exopolysaccharide by *Antrodia camphorata* in batch cultures. Process Biochem..

[23] Fang Q.-H., Zhong J.-J. (2002). Effect of Initial PH on Production of Ganoderic Acid and Polysaccharide by Submerged Fermentation of Ganoderma Lucidum. Process Biochem..

[24] Hu S., Xiao X., Wu X., Xia X., Yu Y., Wu H. (2017). Comparative transcriptomic analysis by RNA-Seq of acid tolerance response (ATR) in EHEC O157:H7. LWT-Food Sci. Technol..

[25] Shan K., Wang C., Liu W., Liu K., Jia B., Hao L. (2019). Genome sequence and transcriptomic profiles of a marine bacterium, *Pseudoalteromonas agarivorans* Hao 2018. Sci. Data.

[26] Blackwell J. (1977). Infrared and Raman Spectroscopy of polysaccharides. Extracellular Microbial Polysaccharides.

[27] Deng Y.-Y., Yi Y., Zhang L.-F., Zhang R.-F., Zhang Y., Wei Z.-C., Tang X.-J., Zhang M.-W. (2014). Immunomodulatory activity and partial characterisation of polysaccharides from *Momordica charantia*. Molecules.

[28] Huang K., Li Y., Tao S., Wei G., Huang Y., Chen D., Wu C. (2016). Purification, characterization and biological activity of polysaccharides from *Dendrobium officinale*. Molecules.

[29] Whistler R.L., House L.R. (1953). Infrared spectra of sugar anomers. Anal. Chem..

[30] Yi Y., Liao S.T., Zhang M.W., Shi J., Zhang R.F., Deng Y.Y., Wei Z.C. (2011). Physicochemical characteristics and immunomodulatory activities of three polysaccharide-protein complexes of *Longan* pulp. Molecules.

[31] Wang C.-Z., Zhang H.-Y., Li W.-J., Ye J.-Z. (2015). Chemical Constituents and Structural Characterization of Polysac charides from Four Typical Bamboo Species Leaves. Molecules.

[32] Kačuráková M., Wilson R.H. (2001). Developments in mid-infrared FT-IR spectroscopy of selected carbohydrates. Carbohydr. Polym..

[33] Yang L., Zhang L.-M. (2009). Chemical structural and chain conformational characterization of some bioactive polysaccharides isolated from natural sources. Carbohydr. Polym..

[34] Marijuán P.C., Navarro J., del Moral R. (2010). On Prokaryotic Intelligence: Strategies for Sensing the Environment. Biosystems.

[35] Appleby J.L., Parkinson J.S., Bourret R.B. (1996). Signal transduction via the multi-Step phosphorelay: Not necessarily a road less traveled. Cell.

[36] Jin Q., Yuan Z., Xu J., Wang Y., Shen Y., Lu W., Wang J., Liu H., Yang J., Yang F. (2002). Genome sequence of *Shigella flexneri* 2a: Insights into pathogenicity through comparison with genomes of *Escherichia coli* K12 and O157. Nucleic Acids Res..

[37] Stephenson K., Hoch J.A. (2002). Histidine kinase-mediated signal transduction systems of pathogenic microorganisms as targets for therapeutic intervention. Curr. Drug Targets-Infect. Disord..

[38] Watanabe T., Hashimoto Y., Yamamoto K., Hirao K., Ishihama A., Hino M., Utsumi R. (2003). Isolation and charac terization of inhibitors of the essential histidine kinase, *YycG* in *Bacillus subtilis* and *Staphylococcus aureus*. J. Antibiot..

[39] Bernardini M.L., Fontaine A., Sansonetti P.J. (1990). The two-component regulatory system *OmpR-EnvZ* controls the virulence of *Shigella flexneri*. J. Bacteriol..

[40] Yang J., Wang J., Yao Z.-J., Jin Q., Shen Y., Chen R. (2003). GenomeComp: A visualization tool for microbial genome comparison. J. Microbiol. Methods.

[41] Kumar S., Tamura K., Jakobsen I.B., Nei M. (2001). MEGA2: Molecular evolutionary genetics analysis software. Bioinformatics.

[42] Sun M.-L., Zhao F., Shi M., Zhang X.-Y., Zhou B.-C., Zhang Y.-Z., Chen X.-L. (2015). Characterization and biotechno logical potential analysis of a new exopolysaccharide from the Arctic marine bacterium *Polaribacter* sp. SM1127. Sci. Rep..

[43] Lo T.C.-T., Chang C.A., Chiu K.-H., Tsay P.-K., Jen J.-F. (2011). Correlation evaluation of antioxidant properties on the monosaccharide components and glycosyl linkages of polysaccharide with different measuring methods. Carbohydr. Polym..

[44] Meng L., Sun S., Li R., Shen Z., Wang P., Jiang X. (2015). Antioxidant activity of polysaccharides produced by *Hirsutella* sp. and relation with their chemical characteristics. Carbohydr. Polym..

[45] Satpute S.K., Banat I.M., Dhakephalkar P.K., Banpurkar A.G., Chopade B.A. (2010). Biosurfactants, bioemulsifiers and exopolysaccharides from marine microorganisms. Biotechnol. Adv..

[46] Marzan L.W., Shimizu K. (2011). Metabolic regulation of *Escherichia coli* and its *PhoB* and *PhoR* genes knockout mutants under phosphate and nitrogen limitations as well as at acidic condition. Microb. Cell Factories.

[47] Sultan S.Z., Silva A.J., Benitez J.A. (2010). The *PhoB* regulatory system modulates biofilm formation and stress response in *El Tor Biotype Vibrio Cholerae*. FEMS Microbiol. Lett..

[48] Hickman J.W., Tifrea D.F., Harwood C.S. (2005). A Chemosensory system that regulates biofilm formation through modulation of cyclic diguanylate levels. Proc. Natl. Acad. Sci. USA.

[49] Park S., Kelley K.A., Vinogradov E., Solinga R., Weidenmaier C., Misawa Y., Lee J.C. (2010). Characterization of the structure and biological functions of a capsular polysaccharide produced by *Staphylococcus saprophyticus*. J. Bacteriol..

[50] Shang N., Xu R., Li P. (2013). Structure characterization of an exopolysaccharide produced by *Bifidobacterium animalis* RH. Carbohydr. Polym..

[51] Papageorgiou S.K., Kouvelos E.P., Favvas E.P., Sapalidis A.A., Romanos G.E., Katsaros F.K. (2010). Metal–Carboxylate interactions in metal–alginate complexes studied with FTIR spectroscopy. Carbohydr. Res..

[52] Guo L., Zhu W., Xu F., Liu M., Xie Y., Zhang J. (2014). Optimized ultrasonic-assisted extraction of polysaccharides from *Cyclina sinensis* and evaluation of antioxidant activities in vitro. CyTA-J. Food.

[53] Liu S., Ai Z., Qu F., Chen Y., Ni D. (2017). Effect of steeping temperature on antioxidant and inhibitory activities of green tea extracts against α-Amylase, α-Glucosidase and intestinal glucose uptake. Food Chem..

[54] Liao W., Luo Z., Liu D., Ning Z., Yang J., Ren J. (2015). Structure characterization of a novel polysaccharide from *Dictyophora Indusiata* and its macrophage immunomodulatory activities. J. Agric. Food Chem..

[55] Martin M. (2011). Cutadapt removes adapter sequences from high-throughput sequencing reads. EMBnet. J..

[56] Langmead B., Salzberg S.L. (2012). Fast Gapped-Read alignment with Bowtie 2. Nat. Methods.

[57] Kim D., Pertea G., Trapnell C., Pimentel H., Kelley R., Salzberg S.L. (2013). TopHat2: Accurate alignment of transcriptomes in the presence of insertions, Deletions and Gene Fusions. Genome Biol..

[58] Anders S., Pyl P.T., Huber W. (2015). HTSeq—A python framework to work with high-throughput sequencing data. Bioinformatics.

[59] Wang L., Wang S., Li W. (2012). RSeQC: Quality control of RNA-Seq experiments. Bioinformatics.

[60] Ashburner M., Ball C.A., Blake J.A., Botstein D., Butler H., Cherry J.M., Davis A.P., Dolinski K., Dwight S.S., Eppig J.T. (2000). Gene Ontology: Tool for the unification of biology. Nat. Genet..

[61] Kanehisa M., Goto S. (2000). KEGG: Kyoto encyclopedia of genes and genomes. Nucleic Acids Res..

