# Supplementary material for: Effect of Different Initial Fermentation pH on Exopolysaccharides Produced by Pseudoalteromonas agarivorans Hao 2018 and Identification of Key Genes Involved in Exopolysaccharide Synthesis via Transcriptome Analysis
Source: Mar Drugs. 2022 Jan 20;20(2):89. doi: 10.3390/md20020089 (PMC8877158; doi:10.3390/md20020089)
Supplement: Supplementary file 1 [file marinedrugs-20-00089-s001.zip › marinedrugs-1507914 supplementary.pdf]

Effect of Different Initial Fermentation pH on Exopolysaccharides Produced by *Pseudoalteromonas agarivorans* Hao 2018 and Identification of Key Genes Involved in Exopolysaccharide Synthesis via Transcriptome Analysis

Yuhao Ju [1], Kai Shan [1], Wenlin Liu [1], Chenxiang Xi [1], Yiling Zhang [1], Wei Wang [1], Chunlei Wang [1], Ruiwen Cao [1], Wenxing Zhu [1], Haiyong Wang [1], Yanqiu Zhao [1] and Lujiang Hao [1,2,*]

[1] School of Bioengineering, Qilu University of Technology (Shandong Academy of Sciences), Jinan 250353, China; 10431201111@stu.qlu.edu.cn (Y.J.); kai_shan@163.com (K.S.); 1043118323@stu.qlu.edu.cn (W.L.); 1043119587@stu.qlu.edu.cn (C.X.); 10431200980@stu.qlu.edu.cn (Y.Zhang); 10431211258@stu.qlu.edu.cn (W.W.); chunlei_wang79@163.com (C.W.); rwcao@qlu.edu.cn (R.C.); wenxingzhu@qlu.edu.cn (W.Z.);
wangchemical@126.com (H.W.); 10431211120@stu.qlu.edu.cn (Y.Zhao)

[2] State Key Laboratory of Biobased Material and Green Papermaking, Qilu University of Technology
(Shandong Academy of Sciences), Jinan 250353, China

* Correspondence: lujiang_hao@qlu.edu.cn

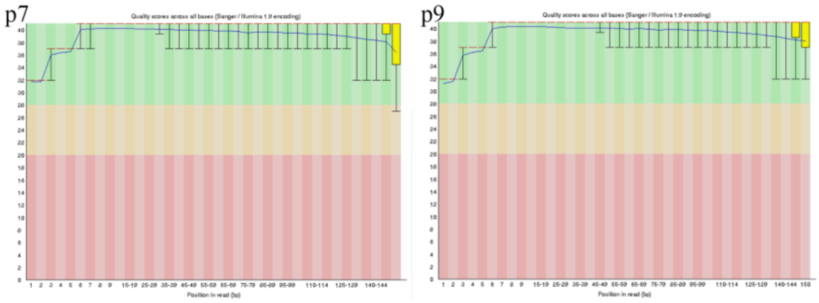

Figure S1. Single base mass distribution chart.

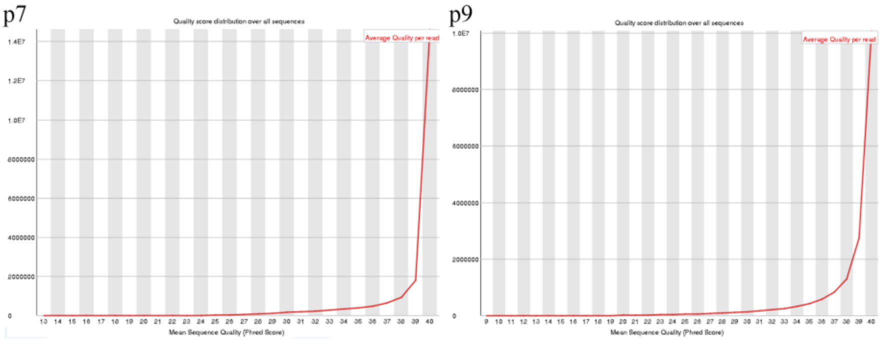

Figure S2. Reads average mass distribution (partial).

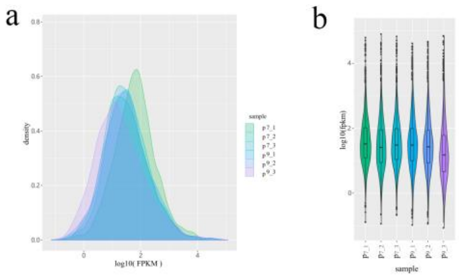

Figure S3. FPKM density distribution and density distribution statistics: (a) FPKM density distribution, (b) FPKM density distribution statistical.

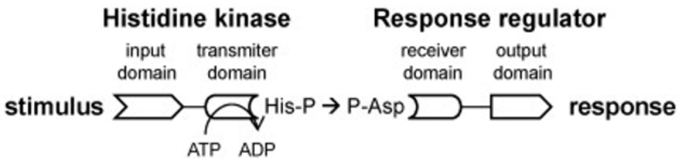

Figure S4. Regulatory mechanism of the two-component system.

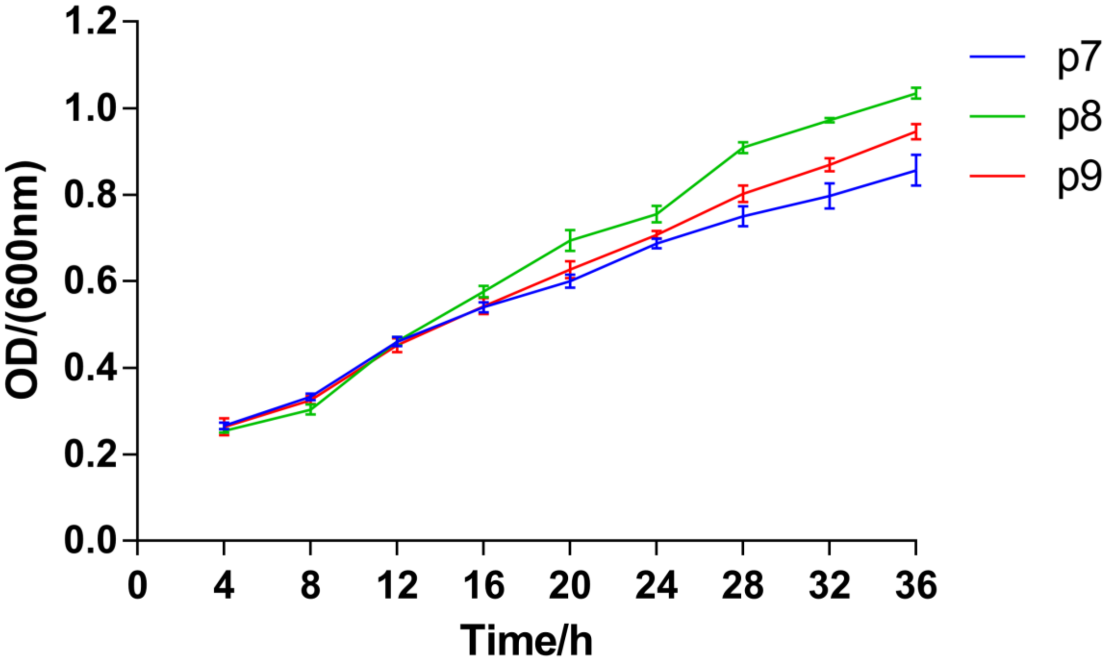

Figure S5. The growth curves of *P. agarivorans* Hao 2018 under different initial fermentation pH conditions.

Table S1. Culture conditions of *P. agarivorans* Hao 2018

Environmental Factors	Level
Fermentation initial pH	7
	8
	9

Table S2. Results of GO enrichment analysis

GO ： ID	Term	Category	DEG
GO:0060089	molecular transducer activity	MF	118
GO:0004872	receptor activity	MF	84
GO:0004871	signal transducer activity	MF	88
GO:0007165	signal transduction	BP	91
GO:0023052	signaling	BP	91
GO:0007154	cell communication	BP	95
GO:0007049	cell cycle	BP	25
GO:1901293	nucleoside phosphate biosynthetic process	BP	72
GO:0009165	nucleotide biosynthetic process	BP	71
GO:0000156	phosphorelay response regulator activity	MF	62
GO:0090407	organophosphate biosynthetic process	BP	91
GO:0016881	acid-amino acid ligase activity	MF	11
GO:0000160	phosphorelay signal transduction system	BP	72
GO:0018130	heterocycle biosynthetic process	BP	203
GO:0035556	intracellular signal transduction	BP	77
GO:0034654	nucleobase-containing compound biosynthetic process	BP	168
GO:0019438	aromatic compound biosynthetic process	BP	195
GO:0038023	signaling receptor activity	MF	54
GO:0040011	locomotion	BP	29
GO:0009279	cell outer membrane	CC	46

Table S3. Expression of EPS transporter-related genes

Annotation	Gene ID	p7_FPKM	p9_FPKM
glucose/galactose MFS transporter	chr1_1825	5.12	124.45
glucose/galactose MFS transporter	chr2_538	14.25	245.99
Glucose transport protein	chr1_1082	21.33	143.80

Table S4. Expression of genes related to nucleotide sugar synthesis

Annotation	Gene ID	p7_FPKM	p9_FPKM
glucokinase	chr1_1425	34.80	30.35
phosphoglucomutase	chr1_1667	405.09	99.66
dTDP-glucose 4,6-dehydratase	chr1_421	101.41	68.63
dTDP-4-dehydrorhamnose 3,5-epimerase	chr1_419	203.74	114.49
dTDP-4-dehydrorhamnose reductase	chr1_420	154.70	108.52
UTP--glucose-1-phosphate uridylyltransferase	chr1_1334	194.17	82.68
UTP--glucose-1-phosphate uridylyltransferase	chr1_2358	217.13	112.20
UTP--glucose-1-phosphate uridylyltransferase	chr1_459	2972.63	1595.51
UDP-glucose 6-dehydrogenase	chr1_460	146.31	53.86
UDP-glucuronate-epimerase	chr1_409	31.66	16.85
phosphomannomutase	chr1_423	54.86	170.44
mannose-1-phosphate guanylyltransferase	chr1_422	59.74	145.72

Table S5. Expression of genes related to glycosyltransferase synthesis

Annotation	Gene ID	p7_FPKM	p9_FPKM
glucosyl transferase family 2	chr1_1341	9.34	17.94
Alpha-1,4-glucan:maltose-1-phosphate maltosyltransferase (Fragment)	chr1_1421	11.56	16.50
glycosyltransferase	chr1_309	839.02	251.11
glucosyl transferase family 2	chr1_3148	6527.51	8377.47
glycosyl transferase family 2	chr1_406	14.28	4.33
glycosyl transferase family 1	chr1_440	36.24	40.54
glycosyl transferase family 1	chr1_444	44.36	33.16
gycosyl transferase family 2	chr1_446	27.69	20.22
glycosyl transferase	chr1_449	51.19	23.85
glycosyl transferase	chr1_450	46.22	35.30
rhamnosyltransferase	chr1_451	11.13	15.61
glycosyl transferase	chr1_659	935.43	1295.93
glycosyl hydrolase family 16	chr2_178	43.92	230.86
glycosyl transferase	chr2_200	18.73	22.60
glycosyl transferase	chr2_415	17.07	15.70
glycosyl transferase family 25	chr2_502	1981.20	209.22

Table S6. Effect of different initial fermentation pH on the yield of crude EPS

Environmental Factors	Conditions	EPS crude product yield ($g \cdot L^{-1}$)
Initial pH	7	16.62±0.21
	8	19.34±0.16
	9	18.34±0.02
